# The Flowering Season-Meter at FLOWERING LOCUS C Across Life Histories in Crucifers

**DOI:** 10.3389/fpls.2021.640442

**Published:** 2021-03-11

**Authors:** Diana Mihaela Buzas, Haruki Nishio, Hiroshi Kudoh

**Affiliations:** ^1^Faculty of Life and Environmental Sciences, Tsukuba-Plant Innovation Research Center, University of Tsukuba, Tsukuba, Japan; ^2^Center for Ecological Research, Kyoto University, Otsu, Japan

**Keywords:** Flowering Locus C, polycomb and trithorax chromatin regulators, bistable states, annual and perennial life history, convergent cross mapping

## Abstract

Many plant species overwinter before they flower. Transition to flowering is aligned to the seasonal transition as a response to the prolonged cold in winter by a process called vernalization. Multiple well-documented vernalization properties in crucifer species with diverse life histories are derived from environmental regulation of a central inhibitor of the flowering gene, *Flowering Locus C* (*FLC*). Episode(s) of flowering are prevented during high *FLC* expression and enabled during low *FLC* expression. *FLC* repression outlasts the winter to coincide with spring; this heterochronic aspect is termed “winter memory.” In the annual *Arabidopsis thaliana*, winter memory has long been associated with the highly conserved histone modifiers Polycomb and Trithorax, which have antagonistic roles in transcription. However, there are experimental limitations in determining how dynamic, heterogenous histone modifications within the *FLC* locus generate the final transcriptional output. Recent theoretical considerations on cell-to-cell variability in gene expression and histone modifications generating bistable states brought support to the hypothesis of chromatin-encoded memory, as with other experimental systems in eukaryotes. Furthermore, these advances unify multiple properties of vernalization, not only the winter memory. Similarly, in the perennial *Arabidopsis halleri* ssp. *gemmifera*, recent integration of molecular with mathematical and ecological approaches unifies *FLC* chromatin features with the all-year-round memory of seasonal temperature. We develop the concept of FLC season-meter to combine existing information from the contrasting annual/perennial and experimental/theoretical sectors into a transitional framework. We highlight simplicity, high conservation, and discrete differences across extreme life histories in crucifers.

## Introduction

As a standing proof of a sharp divide between winter and spring, some plant species burst into flower at this precise time to secure reproductive success in this vacant niche. The process of vernalization, enabling precision in timing of the floral transition after the prolonged cold of winter, is versatile in nature and agriculture. Species with single and multiple reproductive episodes (herein annual and perennial), ecotypes adapted to local climates, including those of the model plant species *Arabidopsis thaliana* (*A. thaliana*) from the representative crucifer family, and crop varieties with distinct timing for harvesting, all overwinter before they flower. How can reproductive transition be so precisely timed to occur in spring in a variety of biological systems?

In crucifers, central to vernalization is a gene that blocks flowering termed *Flowering Locus C* (*FLC*) (Michaels and Amasino, 2000). Across life histories, abundant *FLC* expression facilitates vegetative growth until, under the influence of low temperatures, *FLC* expression gradually becomes negligible by the end of winter. Also, as *FLC* repression outlasts the winter, plants become competent to flower in spring—this is often referred to as “winter memory.” The length of winter memory, i.e., the length of time the low FLC expression is maintained, varies especially across extreme life histories: in the annual *A. thaliana*, the *FLC* minimum is prolonged into the final senescence stage; ancestral perennial forms have short winter memory, allowing only a narrow window of flowering before reverting to vegetative growth based on high *FLC* levels (Wang et al., 2009; Aikawa et al., 2010).

Intensive genetic screens dissecting winter memory in *A. thaliana* frequently recover core and accessory proteins of the Polycomb group (PcG) and Trithorax group (TrxG) complexes (reviewed in Buzas et al., 2012; Berry and Dean, 2015a; Luo and He, 2020), highly conserved in eukaryotes (Steffen and Ringrose, 2014). PcG/TrxG catalyze mainly trimethylation of lysine 27 and four of histone 3 (H3K27me3, H3K4me3), while other associated activities, including, for example, H3K36me3, also mediate PcG/TrxG regulated processes (Steffen and Ringrose, 2014). There are two main long-standing conundrums in this area. One is whether (PcG/TrxG induced) chromatin modifications produce memory (Moazed, 2011). The other is how to unify the well-known role of PcG/TrxG in stably maintaining expression states independent of the inducing signals at a gene, with highly dynamic regulation at other PcG/TrxG target genes (Steffen et al., 2012; Steffen and Ringrose, 2014; Reinig et al., 2020). From theoretical consideration on how cell-to-cell variability in gene expression and histone modifications influence the vernalization process, recent advances support the hypothesis of chromatin coding of memory (Angel et al., 2011; Berry et al., 2015b; Yang et al., 2017; Nishio et al., 2020a) as we outline in the section “*Insights From Annual Vernalization: Experiments and Theory*” and in the section “*Initial Insights From Perennial Vernalization*”. By comparing winter memory mechanisms in annuals and perennials, we formulate a model of how stable and flexible gene expression states residing together at *FLC* may account for precise detection of seasons in diverse life histories (see section “*The Flowering Locus C Season-Meter for Flowering Across Life Histories*”). As such, findings from the vernalization example are more generally relevant to chromatin regulation.

## Insights From Annual Vernalization: Experiments and Theory

### Models for *Arabidopsis thaliana*

Establishing a gene expression state at the chromatin level involving PcG/TrxG was initially determined experimentally with the Hox genes in *Drosophila* as a two-step process: initiation and maintenance. Initiation commonly requires DNA-based mechanisms, mediated by transcription factor binding. Maintenance over cell generations is under the influence of histone modifications, recruited locally by *cis*-acting elements termed Polycomb/TrxG recruiting elements (PRE/TRE) (Steffen and Ringrose, 2014; Buzas, 2017). Essentially memory elements PRE/TREs can maintain a high degree of stability of gene expression across cell divisions that outlast the initiating signals. The states maintained as development proceeds are either ON or OFF; this can be visualized in reporter assays as variegation (Chan et al., 1994; Steffen et al., 2012).

Theoretical approaches also bridge PcG/TrxG-related phenomena across eukaryotes and are deemed suitable to tackle the complex dynamics, especially in quantitative terms (Steffen et al., 2012). How an array of nucleosomes can remain stable over many cell divisions even when individual nucleosomes change modification states randomly has been predicted from a purely theoretical study (Dodd et al., 2007). In this model, various histone-modifying biochemical activities result in nucleosome states, distilled down to three categories: “active,” “unmodified,” and “repressed.” The transitions between these states can be *via* either feedback (a nucleosome state generates the same state in neighboring nucleosomes) or noise (random addition or removal of modifications). The model asks what conditions generate bistability, a state where the whole array of nucleosomes is either ON or OFF, giving rise to variegation (Chan et al., 1994; Steffen et al., 2012). Feedback is required for system stability, while noise generates switching. How the Dodd model is applicable to vernalization (Angel et al., 2011; Satake and Iwasa, 2012) is outlined in the section “*Cell-to-Cell Variation Underlies Measuring of Cold Duration, Robust Response to Noisy Temperatures, and Memory of Winter.*”

### The Repertoire of Stable *AtFLC* Expression States

Both active and repressed states of *AtFLC* are biologically relevant: active *AtFLC* reliably prevents flowering until plants have experienced winter/saturating cold treatment, while repressed *AtFLC* ensures that flowering takes place in spring/after cold treatment. How each of the active and the repressed *AtFLC* states are established has been investigated as two-step processes, as in *Drosophila*, especially as *AtFLC* regulatory regions satisfying both PRE and TRE requirements have been found (reviewed in Buzas et al., 2012).

When considering how active *AtFLC* is established, the two steps can be delineated: a transcription factor transiently expressed in embryos initiates *AtFLC* activation; then, this memory of the embryonic state is maintained *via* TrxG-like activity during the vegetative phase (Tao et al., 2017). However, the process of *AtFLC* repression does not entirely fit the paradigm. A transcription factor transiently induced in the early stages of cold that would initiate *AtFLC* repression has not been identified (Helliwell et al., 2015). Nevertheless, an early cold event leads to rapid reduction in *AtFLC* transcription rate (Finnegan, 2015). Then, a dynamic cascade of chromatin events during early stages of cold leads to stabilization of *AtFLC* repression over time (Berry et al., 2015b; Finnegan, 2015; Qüesta et al., 2016; Yuan et al., 2016; Yang et al., 2017) (also see section The cell autonomous chromatin switch), before *AtFLC* repression is maintained in a PcG-dependent manner (Buzas et al., 2012; Berry and Dean, 2015a).

Adding support to the PcG/TrxG control *via* bistable states, *AtFLC* ON and OFF states have been evidenced experimentally (Angel et al., 2011; Berry and Dean, 2015a; Yang et al., 2017; Qüesta et al., 2020). However, surprisingly, *AtFLC* ON/OFF states are not exclusively found in developmental windows of active/repressed *AtFLC*. Instead, mixed ON/OFF states are present during the transition from active to repressed *FLC* (Angel et al., 2011; Berry et al., 2015b; Yang et al., 2017) as well as possibly during the reverse transition (Qüesta et al., 2020). In fact, the transition intervals support further *AtFLC* biological functions: an accelerated increase (*FLC* dial-up) of *AtFLC* levels in the seed both resets the vernalization requirement in the next generation (Sheldon et al., 2008; Choi et al., 2009; Crevillén et al., 2014), and ensures that flowering is prevented (Sheldon et al., 2008); gradually decreasing *AtFLC* (*FLC* dial-down) levels results in corresponding degrees of earliness in flowering (Michaels and Amasino, 2000; Sheldon et al., 2006).

In conclusion, current data prompts a view of the *AtFLC* locus as a dynamic PcG/TrxG target where switching between ON and OFF states generates a cyclic response with four types of *AtFLC* quantities, tightly regulated over time: minimum, dial-up, maximum, and dial-down.

### *AtFLC* Chromatin: Three Domains

It is of great interest to understand how the PcG/TrxG-induced chromatin modifications control *FLC* expression. The whole tissue level dynamics of H3K27me3/H3K4me3 has been quantified at the di-tri nucleosome level resolution along the FLC chromatin during vernalization treatments in many *A. thaliana* studies (Finnegan and Dennis, 2007; Angel et al., 2011; Buzas et al., 2011; Yang et al., 2017). A group of over 30 nucleosomes of the *AtFLC* locus appears to be structured in three regions with distinct H3K4me3/H3K27me3 dynamics (Finnegan and Dennis, 2007; Yang et al., 2014). We note that all three domains can also be distinguished in *A. halleri* (Nishio et al., 2020a,b). The few nucleosomes spanning the short first exon and part of the first intron form a chromatin domain termed the nucleation region, NR. The greater bulk of nucleosomes along the coding region, including the long first intron, which is generally termed the gene body (GB). Finally, the distal end of *FLC*, encompassing the promoter and the 5′ end of the antisense non-coding RNA *COOLAIR* (Swiezewski et al., 2009), marks the third domain, later termed distal Nucleation Region (dNR) (Nishio et al., 2020a,b).

In Table 1, we summarize H3K27me3/H3K4me3 dynamics during *A. thaliana* vernalization treatments for NR, GB, and dNR at the whole tissue level. Intuitive deductions on how distinct dynamics at each/all three chromatin domains input into an overall *AtFLC* transcriptional outcome under different temperature regimes are difficult to make, even when further genetic and biochemical experimental evidence is considered.

**TABLE 1:** The whole tissue level quantities of gene expression and histone modifications from *At* vernalization treatments and yearly *Ahg* in the natural environment.

	***FLC* minimum**		***FLC* dial-up**		***FLC* maximum**		***FLC* dial-down**		
NR	m		–	⇑	M		⇓		H3K4me3
	M		–	M	m		⇑		H3K27me3

GB	M	⇑	–	M	m		m	⇑	H3K27me3

dNR	⇑	m	–	m	⇑	m	M	⇑⇓	H3K4me3
	M	m	–	⇑⇓	m		⇑	m	H3K27me3
	*At*	*Ahg*	*At*	*Ahg*	*At*	*Ahg*	*At*	*Ahg*	

*Flowering locus *C* (*FLC)* expression is divided into minimum, dial-up, maximum, and dial-down. H3K4me3 and H3K27me3 levels are indicated, as follows: m, minimum; M, maximum; ⇑, increasing; ⇓, decreasing. Data are from Yang et al. (2014) for *Arabidopsis thaliana (At*) and Nishio et al. (2020a, b) for *Arabidopsis halleri* (*Ahg*). Quantifications of histone modification data during *AtFLC* dial-up in young embryo are not available, which is denoted as “–.” NR, Nucleation Region; dNR, distal Nucleation Region; GB, gene body.*

### Cell-to-Cell Variation Underlies Measuring of Cold Duration, Robust Response to Noisy Temperatures and Memory of Winter

One way to advance the chromatin profiles outlined in Table 1 to the next level for understanding vernalization characteristics is to move to single nucleosome and single cell level experiments. The immediate alternative is to advance from tissue to cell level based on theoretical predictions and then devise suitable tests with currently available techniques. Two studies have adapted the Dodd model to stochastically implement the chromatin dynamics described in Table 1 for NR and GB in *A. thaliana* by including additional parameters specific to vernalization (temperature-dependent replication rates, etc.) and have simulated these numerically to reproduce the chromatin dynamics observed (Angel et al., 2011; Satake and Iwasa, 2012). Outcomes have been reviewed in more detail (Berry and Dean, 2015a), as well as in more general terms on how epigenetics contributes to quantitative biology (Steffen et al., 2012).

One merit of these models is that they reveal the simplest way to unify the multiple characteristics of the vernalization system, previously dissected molecularly on a one-by-one basis. When the underlying assumption is that the chromatin status links with transcriptional outcome to give memory (H3K27me3 correlates with repression), at least three characteristics of the vernalization system are generated. First, when each cell is in either ON or OFF FLC state and the timing of transition between states is not synchronized in all cells, the length of cold can be measured at the population level as the proportion of cells in the OFF state. For this, strong cooperativity of histone modifications is required. Second, as a warm-to-cold temperature shift triggers de-synchronized switching in individual cells, a robust response to noise is realized at the cell population level (Satake and Iwasa, 2012). Third, to understand how full *FLC* repression can outlast the cold interval Satake and Iwasa (2012) explicitly addressed the duration of winter memory. They found that the speed of deposition of repressive histone modifications after vernalization is critical. The prediction is that the length of winter memory in annuals is large, essentially irreversible, based on high deposition speed, while in perennials, comparatively slower deposition creates shorter winter memory. This also implies that a plant can respond to diverse intervals of cold by adjusting the speed of deposition of repressive marks (Satake and Iwasa, 2012).

The first critical experimental support for these characteristics came from assays in vernalized root cells. Plants expressing a translational *FLC:GUS* fusion exposed to non-saturating vernalization treatment, then transferred to warm, displayed variegated, all-or-nothing *FLC:GUS* expression sectors reflecting the stochastic nature of silencing and re-activation (Angel et al., 2011).

### The Cell-Autonomous Chromatin Switch

A frequent hypothesis is that histone modifications maintain transcription states, i.e., they encode memory. However, a reciprocal relationship between transcription and H3K27me3 accumulation was demonstrated at *AtFLC* (Buzas et al., 2011). Recent studies have shown that *AtFLC* alleles in ON and OFF states co-exist within the same cell in plants that had been vernalized (Berry et al., 2015b; Yang et al., 2017; Qüesta et al., 2020). This indicates a cell autonomous chromatin switch functions during the quantitative stage in the cold (Berry et al., 2015b). There is also additional evidence from the *Lov-1* ecotype of *A. thaliana* where *AtFLC* re-activation occurs, that re-activation is also a cell-autonomous process (Qüesta et al., 2020).

## Initial Insights From Perennial Vernalization

### Perennial Life Cycle

Perennial crucifers cycle through episodes of vegetative and reproductive growth (Wang et al., 2009; Aikawa et al., 2010; Kemi et al., 2013). Genetic studies in the *Arabis alpina* perennial support the role of the FLC ortholog *PERPETUAL FLOWERING 1* in preventing flowering before vernalization based on high *PEP1* expression and restricting flowering to a short episode, before PEP1 reverts to high levels in some meristems (Wang et al., 2009). The perennial life cycle in *Arabidopsis halleri* ssp. *gemmifera* (*A. halleri*) is unique in that all developing meristems first become reproductive, then revert to the vegetative phase by developing aerial rosettes, which then propagate clonally (Aikawa et al., 2010; Satake et al., 2013; Nishio et al., 2016, 2020a,b; Honjo and Kudoh, 2019). These characteristics confer experimental advantages, especially in studies in the natural environment. As *AhgFLC* expression is monitored over an entire year, chromatin events are less rapid than in vernalization treatments and can be captured in slow motion (Nishio et al., 2020a). Particularly, the *FLC* dial-up, restricted to embryonic cells concealed in tiny seeds in *A. thaliana* and to some meristems in *A. alpina*, is represented in readily accessible *A. halleri* tissues.

### Theoretical and Experimental Approaches in *Arabidopsis halleri*

Frequent, long-term time series of gene expression and histone modifications, combined with environmental data in a natural *A. halleri* population (Nishio et al., 2020a) enabled application of two main types of parameter estimations and mathematical models. First, linear regressions were applied assuming the *AhgFLC* expression follows the amount of chilling accumulation below a certain threshold temperature for a set period (Aikawa et al., 2010; Nishio et al., 2020a). This approach allowed us to estimate the length of past exposure to low temperature affecting the current gene expression (L). Second, an empirical dynamic modeling test called convergent cross-mapping (CCM) was used. This test can be applied on multiple data series, when each has more than 30–40 time points (Sugihara et al., 2012; Ye et al., 2015). CCM examines causality between variables by testing whether the information of a casual variable is encoded in the time series of an affected variable. For example, when a variable X affects another variable Y, the signature of X can be found in the time-series of Y. Thus, it is possible to predict the dynamics of X using those of Y, which is called cross-mapping. This is applicable even when there are two-way causal relationships between the elements. CCM was used to infer the causal relationships between H3K4me3 and H3K27me3 at different locations along the *AhgFLC* locus and between mRNA and H3K4me3/H3K27me3 (Nishio et al., 2020a).

### All-Year-Round Memory: Seasonal Temperature and Repertoire of Stable *AhgFLC* States

Yearly dynamics of *AhgFLC* expression revealed a cyclic pattern with maximum, dial-down, minimum, and dial-up quadrants (Aikawa et al., 2010; Nishio et al., 2020a). The transitions between quadrants were delayed relative to the break of seasons (Kudoh, 2016). Indeed, maximum likelihood analysis in simple chilling unit models between temperature and *AhgFLC* expression in a natural habitat revealed that the expression level is determined by temperature during the past period of 42 days, i.e., *L* = 42 days (Aikawa et al., 2010). Furthermore, past temperature also determined the accumulation of histone modifications: H3K27me3 appeared to index the long-term seasonal trend along the *AhgFLC* locus (Nishio et al., 2020a). These results indicate that *AhgFLC* serves as a memory of seasonal temperature throughout the year, not just during the cold season and that all four *AhgFLC* quadrants manifest stability characteristic of PcG/TrxG states. Notably, stability of *AhgFLC* dial-up and dial-down states was supported further: intermediate *AhgFLC* dial-up levels are maintained when plants were transplanted to cold (Nishio et al., 2020a), and the *AhgFLC* dial-down levels are proportional with the length of cold treatments (Nishio et al., 2020b), as known from *A. thaliana* (Sheldon et al., 2006) and *Arabis alpina* (Wang et al., 2009).

### Causal Relationships Between H3K27me3 and Transcription in *Arabidopsis halleri*

The seasonal dynamics of H3K4me3 and H3K27me3 in the natural environment at the three *AhgFLC* chromatin domains is outlined in Table 1. Remarkably similar with the data from *A. thaliana* vernalization treatments are dynamics at the NR region. As this region plays a critical role in the cell autonomous chromatin switch in *A. thaliana* (Angel et al., 2011; Berry et al., 2015b; Yang et al., 2017), it was of interest to interrogate the causal relationship between H3K27me3/H3K4me3 and transcription at the NR in *A. halleri*. The CCM analysis indicated bidirectional causalities at NR between all three pairs: between mRNA and H3K4me3, between mRNA and H3K27me3, and between H3K4me3 and H3K27me3 (Nishio et al., 2020a). Therefore, a causal relationship between transcription and H3K27me3 at NR in *A. halerii* is consistent with the cell autonomous chromatin switch from *A. thaliana* operating in *A. halleri* as well (Nishio et al., 2020a).

## The Flowering Locus C Season-Meter for Flowering Across Life Histories

The current state of knowledge on how Pcg/TxG contributes to precision in determining seasonal transition in a variety of crucifers will undoubtedly advance in the future to better bridge across the life histories and between experiment and theory. Today’s exercise is to create a transitional framework to identify conserved mechanisms across annual and perennial life histories in crucifers.

Simplicity in composition and precision of measurements combine in the operating principles of a mechanical watch, which, once set, can run indefinitely with exquisite precision. Similarly, we envisage that an “FLC season-meter” might precisely detect seasons in the natural environment based on the simple yet versatile properties conferred by bistable states supporting the repertoire of stable *FLC* states. Considering that perennial life history is the ancestral form (Kiefer et al., 2017), it is difficult to imagine that bistable states arose in the derivative annual forms.

**FIGURE 1:**
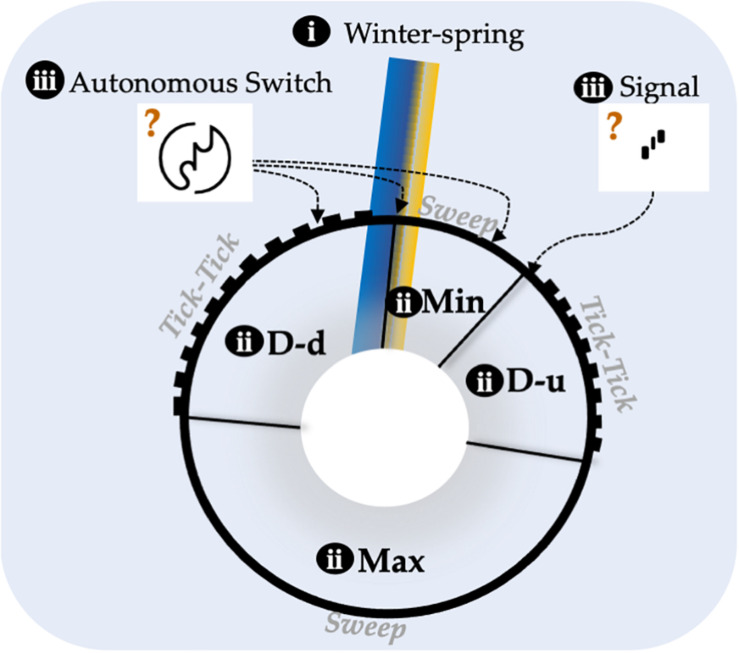
Putative conserved features of the Flowering Locus C (*FLC)* season-meter across annual and perennial crucifers. The cyclic pattern of *FLC* expression is represented as a black circle divided into four quadrants: Max, FLC maximum; Min, FLC minimum; D-d, FLC dial-down; D-u, *FLC* dial-up (ii). The transition D-d-Min is anchored onto the Winter-Spring transition, represented by the blue/yellow (for cold-warm temperatures) gradient rectangular (i). There are two types of inputs into the cyclic response, i.e., the *FLC* season-meter is “hybrid”: autonomous chromatin switch and transcription-factor based signal (iii). The autonomous Chromatin switch generates graded response (tick-tick) quadrants when switching is still taking place and ON/OFF states are mixed and smooth response (sweep) when switching had resolved into full either ON or OFF states. The three arrows from the autonomous switch and one arrow from the signal indicate control points based on Angel et al. (2011); Berry et al. (2015b), Yang et al. (2017), and Tao et al. (2017). The length of each quadrant in the image is approximated from Nishio et al. (2020a) (Max, 50%; D-d 25%, Min 10%, and D-u 15%). Brown question marks indicate that the inputs could be located elsewhere.

Based on arguments outlined in the section “*Insights From Annual Vernalization: Experiments and Theory*” for the annual *A. thaliana* and in the section “*Initial Insights From Perennial Vernalization*” mainly for the perennial *A. halleri*, we propose that the FLC season-meter may have three core principles (Figure 1):

i) It is anchored, i.e., vernalization requirement aligns the dial-down–minimum transition to the winter–spring transition.
ii) It has a cyclic response with four quadrants of *FLC* expression.
iii) It is hybrid, i.e., two types of inputs trigger changes in *FLC* expression: signals generated by external/developmental triggers (transcription factor based) and cell autonomous switches (chromatin based).

In this view, *FLC* is neither a repressed/active PcG/TrxG target gene nor one that simply switches dynamically, but both. Stability and flexibility induced by PcG/TrxG is manifested during all four *FLC* quadrants, although to different extents within and across life histories.

## Final Remarks

The season-meter concept can generate a list of new questions. How does an autonomous switch influence the length of the consecutive sweep quadrant? Do autonomous switches and signal-triggered transitions alternate? Does a season-meter need to contain more than the minimum number of autonomous switches and signal triggers to maintain the noise buffering function? The list goes on. It is difficult to imagine how to engineer a live-cell measurement system that can track *FLC* switching during an entire cycle in a crucifer growing in the natural environment, but experimentalists and theoretical biologists working together can make the task of deciphering the *FLC* season-meter less insurmountable in the near future.

## Author Contributions

DB conceptualized and wrote the manuscript. HN and HK contributed to the drafting of the paragraphs.

## Conflict of Interest

The authors declare that the research was conducted in the absence of any commercial or financial relationships that could be construed as a potential conflict of interest.
